# Evaluating the implementation of the National Primary Health Care Development Agency (NPHCDA) gateway for the Basic Healthcare Provision Fund (BHCPF) across six Northern states in Nigeria

**DOI:** 10.1186/s12913-024-11867-3

**Published:** 2024-11-14

**Authors:** Uchenna Igbokwe, Raihanah Ibrahim, Muyi Aina, Musa Umar, Muhammed Salihu, Efosa Omoregie, Firdausi Umar Sadiq, Benson Obonyo, Rilwanu Muhammad, Salisu Idris Isah, Natsah Joseph, Babagana Wakil, Faruk Tijjani, Abubakar Ibrahim, Mohammed Nura Yahaya, Eric Aigbogun

**Affiliations:** 1 Solina Centre for International Development and Research, 8 Libreville Cres, Wuse, Abuja, Federal Capital Territory 904101 Nigeria; 2 Bill and Melinda Gates Foundation, 45 Aguiyi Ironsi St, Wuse, Abuja, Federal Capital Territory 904101 Nigeria; 3 Bauchi State Primary Health Care Development Agency, Ministry of Health, Bello Kirfi Road, G.R.A, Bauchi, Bauchi State Nigeria; 4 Kaduna State Primary Health Care Development Agency, 78 Tafawa Balewa Road, Kabala Coastain, Kaduna, Kaduna State 800283 Nigeria; 5 Borno State Primary Health Care Development Agency, 1 Mohammed Indimi Road, Maiduguri, Borno State Nigeria; 6 Sokoto State Primary Health Care Development Agency, Block 14, First and Third Floors, Shehu Kangiwa Secretariat, Sokoto, Sokoto State Nigeria; 7 Yobe State Primary Health Care Development Agency, Yobe State Primary Health Care Board, Ministry of Works Complex, Gashua Road, Damaturu, Yobe State Nigeria; 8 Kano State Primary Health Care Development Agency, Na-Ibawa, Kano-Zaria Rd, Kano, Kano State Nigeria

**Keywords:** NPHCDA gateway, BHCPF implementation, Healthcare, Health services, Universal health coverage, Northern Nigeria

## Abstract

**Background:**

This evaluation research utilized both qualitative and quantitative methods to assess the implementation of the National Primary Health Care Development Agency (NPHCDA) gateway of the Basic Health Care Provision Fund (BHCPF) across six states in Northern Nigeria: Bauchi, Borno, Kaduna, Kano, Sokoto, and Yobe.

**Methods:**

This was a mixed-method research that utilized longitudinal surveys and Key informant interviews to gather information about the implementation status of the BHCPF-NPHCDA gateway. Checklists were developed based on the BHCPF’s national guidelines to gather quantitative data, while simple open-ended questionnaires were used to collect qualitative data from the state BHCPF Program Implementation Unit (PIU) focal persons as key informants.

**Results:**

The result revealed that the NPHCDA had accredited these six states to use one Primary Health Care (PHC) facility in each political ward to implement the BHCPF. Factors that contributed to the success achieved in some states included the early completion of contingent start-up activities, well-established coordination structures, strong support from partners, and the availability of established financial management systems. However, the delays in the submission of quarterly business plans by the BHCPF facilities affected timely approval and fund disbursement. Other challenges included staff capacity gaps, inadequate human resources, and poor management and supervision from the state health agency teams.

**Conclusion:**

There was suboptimal implementation of the BHCPF in at least one thematic area across all states. Therefore, actions such as government commitment for improved coordination, continuous capacity building, effective monitoring and evaluation, and targeted supportive supervision using innovative approaches should be undertaken to improve the program’s implementation. In a broader setting, the insights from BHCPF implementation are valuable for LMICs, offering guidance on overcoming implementation challenges associated with PHC financing. This research provides a resource for enhancing healthcare financing strategies in similar contexts.

**Supplementary Information:**

The online version contains supplementary material available at 10.1186/s12913-024-11867-3.

## Introduction

### Background

According to the World Health Organization (WHO) [1], a population’s health can be improved only through deliberate and committed efforts to invest resources in healthcare. This is reflected in the Abuja Declaration of 2001, where African leaders agreed to allocate 15% of their government expenditure to healthcare [2, 3]. However, as of 2020, only five African countries, including Ethiopia, Gambia, Malawi, Rwanda, and South Africa, had achieved this target [3]. This findings underscore a significant gap between the global commitment of governement to the Abuja Declaration and the actual achievement of this target. As of 2020, highlighting the need for increased efforts and commitment across the continent [1]. 

Investment in a population’s health involves the allocation of adequate resources to healthcare provision closest to them, specifically primary healthcare (PHC) [4, 5]. According to the WHO, the fastest route for achieving equitable universal care and improving the population’s health outcomes is through adequate PHC funding [1, 6, 7]. However, recent WHO data indicates that most African governments spend less than 40% of their healthcare expenditure on primary healthcare [3]. 

Different countries’ depending on their development status and health care system have different funding models for their PHCs [8–10]. However, the fundamental purpose is to improve the quality of service delivery as the PHC are the closest to the general population – to obtain universal health coverage (UHC). In low- and middle-income countries (LMICs), where resource constraints are a major challenge, the PHC funding models vary, which reflects diverse healthcare landscapes and financial contexts. For example, Brazil funds its PHC through a mixed financing model that takes into account a capitation method weighted by equity criteria, payment-for-performance of the Family Health teams, and incentives for strategic and priority actions [11]. Thailand’s financing for UHC is predominantly non-contributory, financed by general government taxation. This mode of financing is based on several assumptions [12, 13]. Ethiopia’s health care and financing strategy utilises multiple financing, but among the commonest funding structures are line-item budget, capitation (per capita), and fee-for-services [14]. Rwanda approaches it PHC funding through a community-based health insurance scheme called the mutuelle de santé (mutuelle), which grants financial and administrative autonomy to every district. It introduced the pay-for-performance or results-based financing that rewards providers for achievements on the quality and value of health care [15, 16]. 

Nigeria, on the other hand, addresses its Primary Health Care (PHC) funding through a government initiative established in 2015 known as the Basic Health Care Provision Fund (BHCPF). This fund is dedicated to improving PHC services, infrastructure, and workforce by providing essential financial support. Emphasizing equity, the BHCPF concentrates on reducing out-of-pocket expenses for individuals accessing basic healthcare services. The fund is strategically structured to reinforce healthcare delivery at the community level, with a specific focus on reaching underserved and vulnerable populations across Nigeria [17].

### Overview of the Basic Health Care Provision Fund (BHCPF) and the National Primary Health Care Development Agency (NPHCDA) gateway

The Basic Health Care Provision Fund (BHCPF) was established in response to the National Health Act in Nigeria, which became law in October 2014 after a decade of planning [17]. The Act provides a legal framework for healthcare services and address the poor state of primary healthcare (PHC). Its implementation is well-timed since Nigeria had witnessed some of the worst health outcomes in the world, contributing to over 50% and 60% of global child and maternal deaths, respectively [18, 19], which is partially due to the poor state of primary healthcare services [17]. The BHCPF, mandated by the National Health Act (NHAct), serves as a special financing vehicle to improve primary healthcare by ensuring funds are available for the healthcare system. Its primary goal is to assist Nigeria in achieving Universal Health Coverage (UHC) by upgrading service quality, addressing financial barriers, and providing emergency medical treatment and services [20]. 

Funding for the BHCPF comes from an annual grant of at least 1% of the Consolidated Revenue Fund (CRF) of the Federal Government and funding from other sources, including grants by donors and the private sector. According to the NHAct (2014), the National Primary Health Care Development Agency (NPHCDA) administers 45% of the BHCPF to improve the operational effectiveness of health facilities for quality primary health care delivery; this has been tagged as the ‘NPHCDA gateway’ in this article; the National Health Insurance Scheme (NHIS) manages 48.75% of the BHCPF to provide a Basic Minimum Package of Health Services (BMPHS) to Nigerians; the National Emergency Medical Treatment Committee (NEMTC), established by the National Council on Health, administers 5% of the fund for the management of health emergencies; and lastly, the Nigeria Centre for Disease Control (NCDC) gateway manages 1.25% of the fund for the provision of public health security [20]. 

The NPHCDA gateway, within its scope, uses the BHCPF to enhance primary healthcare services nationwide, focusing on public PHC facilities. The 45% allocation is divided into Decentralized Facility Funding, with 20% for essential drugs, vaccines, and consumables, 15% for maintenance, and 10% for Human Resources in PHC interventions, including 5% for midwives and 5% for Community Health Influencers, Promoters, and Services (CHIPS) [21]. 

There are key activities associated with BHCPF framework. These include planning and preparation, fund disbursement, fund retirement, and governance and coordination. In planning require coordination among state health officials to ensure PHCs were eligible for BHCPF accreditation, conducting facility assessments, and developing accreditation criteria. This was followed by creation of business plans to guide fund disbursement, and putting measure in place to ensure that healthcare facilities can effectively manage the allocated resources, and BHCPF officers are meant to monitor and follow-up to track accreditation progress, development and timely submission of business in line with the BHCPF implementation requirements. All business plans submitted by healthcare facilities in each quarter will usually receive approval after review by the SPHCDA/MB, which is then transmission to the NPHCDA. After approval of business plans by the NPHCDA, the funds will then be disbursed. Quarterly financial reports from accredited healthcare facilities is the prerequisite for the SPHCDA or SPHCMB to receive quarterly disbursements from the NPHCDA and serve as accountability checks in the NPHCDA gateway of the BHCPF.

The governance structure, as per the NHAct [17], involves four payment gateways managing funds under the supervision of the Honorable Minister of Health and Honorable Commissioners for Health at federal and state levels. Coordination structures exist at the subnational level, with the State Oversight Committee providing leadership, the gateway forum ensuring synergy, and the BHCPF Program Implementation Unit (PIU) handling day-to-day implementation at the state level. The State Primary Health Care Management Agency / Management Board (SPHCDA/MB) houses Program Implementation Units (PIUs) headed by Executive Secretaries/Executive Directors (ESs/EDs). Local Government Health Authorities (LGHAs) and Ward Development Committees (WDCs) provide support at the local government and ward levels, respectively, in co-managing PHC facilities and service delivery [7, 20].

### Rationale

The BHCPF’s NPHCDA gateway was launched in May 2019 across all 36 states of Nigeria and the FCT. Although funding and implementation are essential in primary healthcare systems across states in Nigeria, various challenges may hinder complete and optimal implementation, considering that it is in a nascent stage. The existing literature on the BHCPF has only explored topical issues on the political economy of its design [7, 22–26], accountability mechanisms [25], status of fund release and execution at the national level [7, 27], and broader health financing landscape assessments [28]. These literature had provided valuable insights into the structural and procedural aspects of the BHCPF. However, a notable gap exists in the literature, as the focus had predominantly been on the procedural aspects of the initiative, with limited emphasis on evidence-based assessments of its outcomes.

Currently, there is limited literature providing insights into the current status of the National Primary Health Care Development Agency (NPHCDA) gateway implementation for the Basic Health Care Provision Fund (BHCPF) at the subnational level. This dearth of information leaves a significant gap in guidance regarding the performance of the implementation. Such insight is crucial for executives and policymakers who need to make informed decisions and adjustments to optimize the system. Addressing this information gap is vital for ensuring the effective functioning of the NPHCDA gateway implementation and, consequently, the success of the BHCPF at the subnational level.

There are critical gaps in understanding the operational and programmatic functionality of the gateway in states that have fully implemented the BHCPF strategy. The lack of systematic evaluation hinders a thorough assessment of the program’s effectiveness and the potential challenges it might continue to encounter at the subnational level. For Low- and Middle-Income Countries (LMICs) interested in adopting a similar BHCPF strategy to enhance their Primary Health Care (PHC) systems, these insights are invaluable.

Understanding real-world challenges and successes at the subnational level is critical for LMICs anticipating to adopt comparable frameworks. Policymakers and stakeholders involved in health system innovation can use the findings of this study to establish targeted strategies and improve the implementation of similar PHC funding models. This information’s deep insights enable a context-specific approach to healthcare funding, ensuring that implementation is aligned with the peculiarities of each country’s healthcare landscape.

This study aims to address this gap by evaluating the BHCPF-NPHCDA implementation status across six Northern Nigerian states. The goal is to explore the significant challenges, identify program implications, and offer appropriate recommendations.

## Materials and methods

### Study design

This evaluation research utilized a mixed-method (quantitative (checklist) and qualitative (key informant interviews)) to assess the implementation status of the NPHCDA gateway for the Basic Healthcare Provision Funds (BHCPF) across Bauchi, Borno, Kaduna, Kano, Sokoto, and Yobe states in Northern Nigeria. This paper describes the findings on the status of the implementation across the states, and also provides insights into the successes, challenges, and recommendations for program improvement.

### Study setting

This study focused on six Northern Nigerian states, including Bauchi, Borno, Kaduna, Kano, Sokoto, and Yobe. These states have predominantly rural populations with dispersed settlements, with some urban centers. The majority of the population rely on PHC facilities for healthcare services.

These states were involved in the Northern Nigeria Routine Immunization Strengthening Program (NNRISP), a collaborative initiative between the state governments and various partners, including the Bill and Melinda Gates Foundation (BMGF), the Aliko Dangote Foundation (ADF), and, in some instances, USAID; the Foreign, Commonwealth and Development Office (FCDO); UNICEF; Global Fund; and Global Affairs Canada. The program which first kicked off in Kano (2013) and Bauchi (2014), followed by the other four states in 2015, was to enhance the PHC system through a basket-funding mechanism. The intervention established robust leadership and governance structures, provided technical assistance, and ensured effective oversight and accountability within coordinating units.

The BHCPF’s NPHCDA gateway ensures direct transfer of funds to selected ward PHCs, as a form of decentralized facility financing. Implementation in the six states commenced in Q1 2021, with the expectation of operationalizing it in one PHC per ward in each state. Table S1 shows the number of wards and activated BHCPF healthcare facilities (HFs) in each state as of Q1 of 2021.

**Fig. 1:**
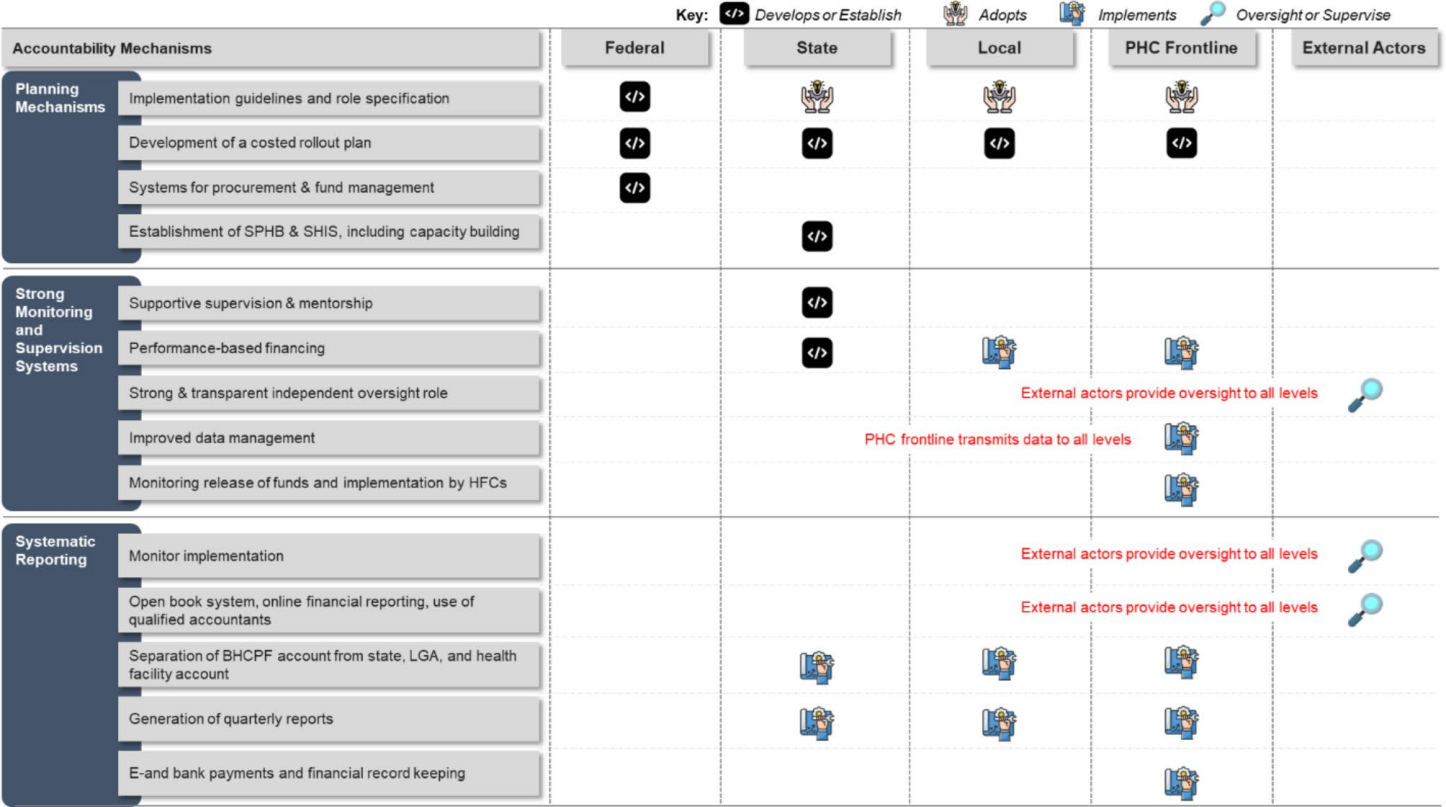
The BHCPF-NPHCDA gateway accountability framework

To ensure proper implementation of the BHCPF’s NPHCDA gateway at all levels, SCIDaR along with the stakeholders developed an accountability framework (See Fig. 1). The framework was then validated and adopted by the BHCPF team of the SPHCDA/MBs of the 6 MoU states. The accountability framework defines clear roles and responsibility of stakeholders of the healthcare system in implementing the BHCPF-NPHCDA gateway at the national and sub-national level. The framework ensures that financial resources are allocated appropriately and in accordance to the goals and priorities set by the guideline, and effective monitoring the financial performance by tracking allocation of funding, expenditures, and adherence to budgetary constraints. Additionally, establishing a governance, resource allocation, and performance monitoring systems, the framework helps detect potential risks and establishes mitigation measures as a proactive safeguarding system against financial impropriety, ensures that the organization operates within established legal and regulatory boundaries, reducing the risk of legal issues. The framework helps establish a basis for continuous improvement in financial management practices through regular reviews and assessments for improved implementation, leading to increased efficiency and effectiveness. This in turn, helps in making informed decision that aligns with broader objectives of the gateway financing structure. Ultimately, this framework helps foster confidence among stakeholders, which includes the population, clients, service providers, donors, and regulatory bodies. When financial management practices are transparent and accountable, it enhances the overall reputation of the health system.

The activities involved in implementing the framework included program planning, fund disbursement, fund retirement, governance and coordination of activities, and supervision of BHCPF-accredited facilities. This study focused on BHCPF-selected PHC facilities across the six states Memorandum of understanding (MoU) states in Northern Nigerian over an 18-month period between January 18 2021 and June 24 2022.

### Data collection tools

#### Quantitative data tool

We developed a checklist using National BHCPF guidelines published by the NPHCDA, which focused on two areas: (1) Implementation status of the BHCPF initiation activities (See Table S2) and (2) Implementation status of routine financial management and governance activities (See Table S3). Initiation activities included the establishment of functional SPHCA/MBs, opening of a Treasury Single Account (TSA) account, evidence of state counterpart funding, identification of one PHC per ward, and subsequent accreditation. The checklist collated data on indicators covering all aspects of the financial cycle, from business planning to retirements and from the onset of implementation to date. The BHCPF focal persons (Director for Planning & Research at the SMH) across the states was useful in providing the status of the activities.

#### Qualitative data tool

Pretested reliable (Cronbach α = 87.2) semi-structured questionnaires as described in Table S4 were developed for key informant interview. The questionnaire was designed to obtain contextual information on the implementation status of activities using physical interviewer-based method [29].

### Data collection methods

We collected quantitative data by conducting desk reviews of program documents. Collaborating with the BHCPF PIU leads in the respective states, teams operating across the six chosen states retrieved and examined relevant documents. These included the states’ planning and accreditation documents, approved quarterly business plans, quarterly disbursement schedules for BHCPF healthcare facilities, payment vouchers, retirement documents submitted by healthcare facilities detailing fund usage, coordination meeting outputs, and supervisory visit schedules and reports. The information extracted from these documents was anticipated to undergo validation processes across various accountability layers, as depicted in Fig. 1, accompanied by rigorous administrative control and restrictions. Consequently, the data obtained is characterized by a minimized level of subjective bias.

For qualitative information, we purposively sampled the six BHCPF PIU leads in each state and conducted interviews with them. These leads were responsible for overseeing the daily implementation of funds at the state level, including the transfer of funds to qualified Primary Health Centers (PHCs), reviewing business plans and reports, managing the retirement submission process, and supervising LGHAs and PHCs. As a result, they possessed first-hand knowledge of the BHCPF implementation program. Although all PIUs were integral members of the implementation team for the BHCPF program, which commenced on January 18, 2021, they were recruited and interviewed just before the project close-out on June 23 2022. This accommodated for changes in any of the PIUs across the state, and for adequate knowledge about the end-to-end processes during implementation. The interviews were conducted face-to-face in English, using semi-structured, pretested (ensuring consistency and reliability in data collection) questionnaire guides. This questionnaire focused on gathering contextual information about the implementation process. The interviews were recorded using a Sony ICD-PX470 Stereo Digital Voice Recorder with a built-in USB connection and were later transcribed verbatim. The transcripts were then shared with the respective PIU leads for validation prior to analysis.

#### Data analysis

Think-cell 2020 (version 11, Fraunhofer venture) was used for the descriptive analysis of the quantitative data, with the results presented as graphs and charts. Additionally, the qualitative data from the Key Informant Interviews (KIIs) were coded using the grounded theory approach for thematic analyses [30, 31]. which involved the use of two research team members to form concepts from the data and independent identification of several themes. The researchers agreed upon the themes and coded open-ended comments for each theme. We evaluated each comment using the constant comparative method of grounded theory [30, 32], then evaluated the entire thematic analysis process using the Clarke and Braun 15-point checklist [33]. The results of the qualitative analysis were visually presented using word map linkages.

## Results

As presented in Table S1, 80.8% (1451/1631) of the functional ward PHCs across the six states evaluated in the study were BHCPF-accredited. All ward PHCs in Bauchi (323; 100%) and Sokoto (244; 100%) received full accreditation for the BHCPF implementation. Ward PHCs in other states received partial accreditation for the BHCPF implementation (Kaduna: 254/255, 99.6%; Borno: 121/147, 82.3%; Yobe: 158/178, 88.8%; Kano: 381/484, 78.7%). The proportion of wards prioritized for the BHCPF in Borno only captured 147 accessible wards of the total 311 wards because of security challenges in other wards.

### Planning and preparation

**Fig. 2:**
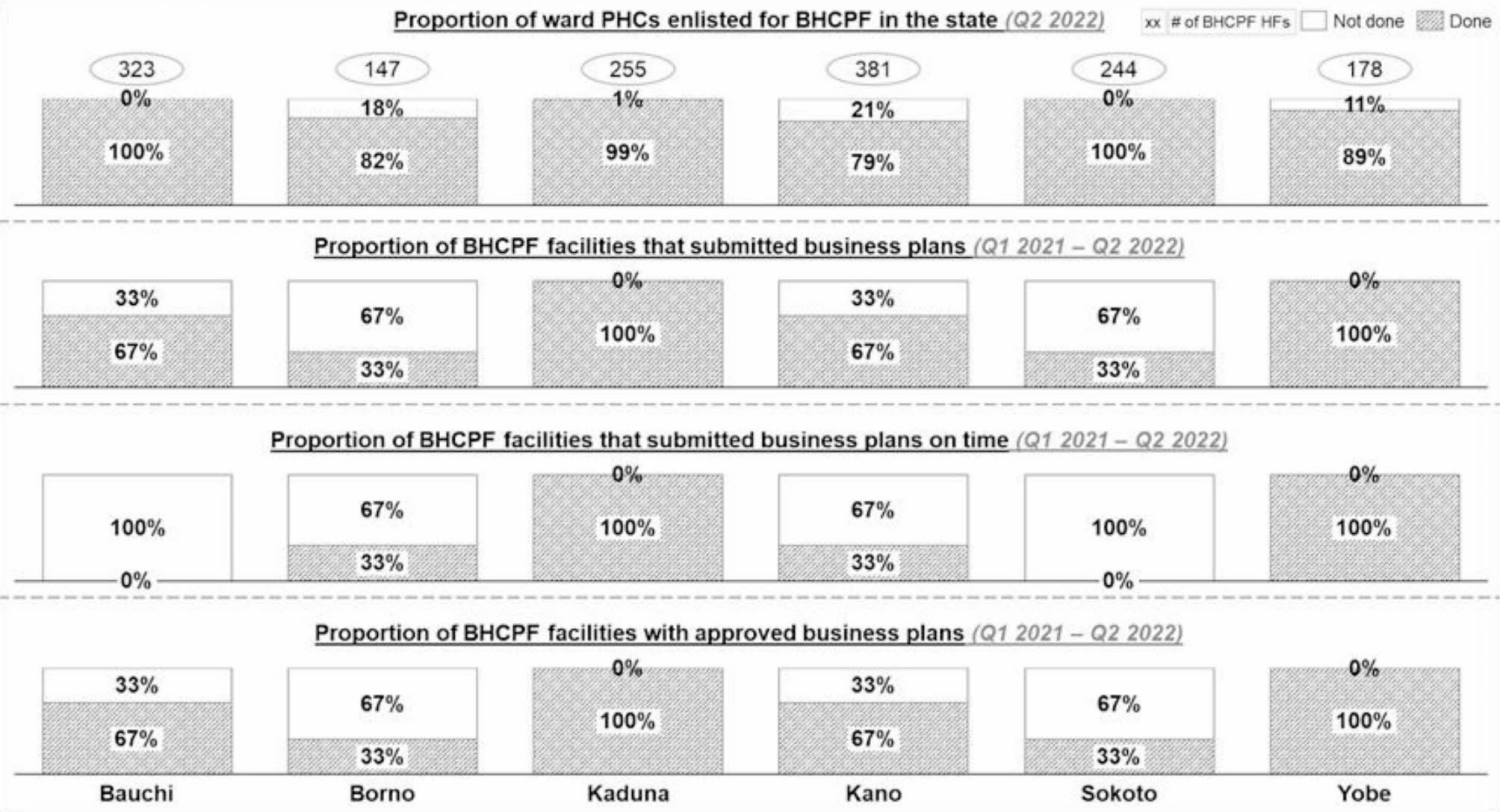
The BHCPF planning activities across the 6 states from Q1 2021 to Q2 2022

As described in Fig. 2, all six states received full/partial accreditation for their eligible ward PHCs to implement the BHCPF. In 2022, the number of PHC facilities at the ward level enlisted for the BHCPF in Sokoto and Yobe had a 26% increase and 2% decrease, respectively. By the beginning of Q1 2022, Bauchi and Sokoto were the only states with a 100% accreditation of one PHC per ward for implementing the BHCPF. After receiving accreditation, ward PHCs developed business plans to guide the disbursement of funds to these healthcare facilities. Five of the six states (except Borno) developed their business plans in Q1 2021. However, only the healthcare facilities in Kaduna and Yobe consistently developed business plans across the six quarters under review. Healthcare facilities in Borno commenced business plan development in Q1 2022. Regarding timeliness, only Kaduna and Yobe consistently submitted business plans on time, while Borno and Kano achieved timely submission in at least 2 quarters.

### Fund disbursement

**Fig. 3:**
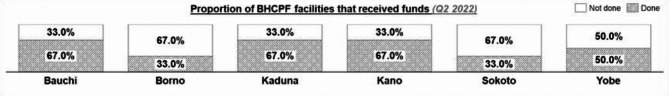
Fund disbursement in the BHCPF across the 6 MoU states from Q1 2021 to Q2 2022

Borno, Kano, and Sokoto disbursed funds for four quarters, while the other three states disbursed funds for two quarters only. Borno and Kano did not commence fund disbursement in Q1 2021 when BHCPF implementation began. Additionally, Kaduna did not disburse funds to 6% (15) of the accredited healthcare facilities (Fig. 3).

### Fund retirement

**Fig. 4:**
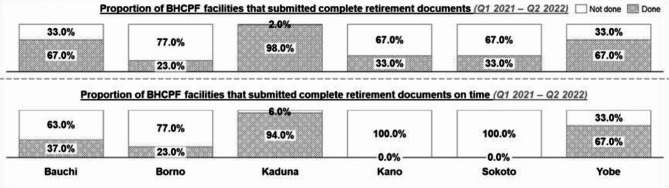
Fund retirement in the BHCPF across the 6 MoU states from Q1 2021 to Q2 2022

Three out of the four states (Bauchi, Sokoto, and Yobe) that disbursed funds to healthcare facilities in Q1 and Q2 2021 successfully retired 100% of funds utilized at the end of the quarters except in Kaduna state, where 94% of healthcare facilities retired the expended funds. Additionally, the retirement of disbursed BHCPF across subsequent quarters was optimal across the states except in Borno (Q1 & Q2 2022), where only 70% of the healthcare facilities submitted retirement documents to the state. According to the National BHCPF guidelines, PHC expenditure statements should be submitted within 15 days of the end of an index quarter. All states except Yobe struggled to achieve the timeline for the retirement of funds as seen in Fig. 4, since this process usually spills over to the next quarter before finalization and submission to the state.

### Governance and coordination

**Fig. 5:**
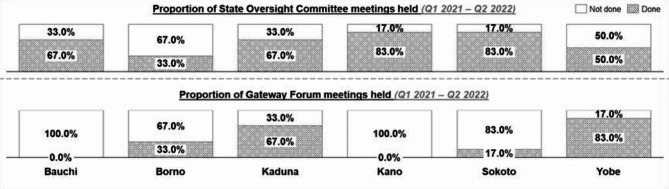
Governance and coordination activities for the BHCPF across the 6 MoU states from Q1 2021 to Q2 2022

**Fig. 6:**
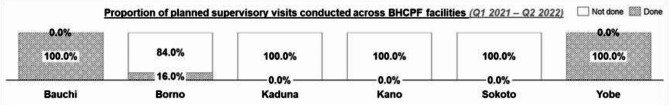
Supervision activities for the BHCPF across the 6 MoU states from Q1 2021 to Q2 2022

All six states reported non-conduct of State Oversight Committee (SOC) meetings in at least one or more quarters from Q1 2022–Q2 2022, as shown in Fig. 5. Only Kano and Sokoto conducted up to five of the six expected meetings over the six reviewed quarters. On the Gateway forum meetings, only four of the six states held at least one of six expected meetings, as seen in Fig. 6. While the remaining two states (Bauchi and Kano) did not convene any gateway forum meetings since the start of the BHCPF implementation.

The SPHCDA/MB is expected to conduct quarterly quality assessments of the PHC facilities; however, only four out of the six states developed supervision plans for the BHCPF healthcare facilities across quarters, with only two states conducting optimal supervision as planned. Kano and Sokoto did not plan or conduct supervisory visits to the BHCPF healthcare facilities for the period under review (Fig. 6).

**Fig. 7:**
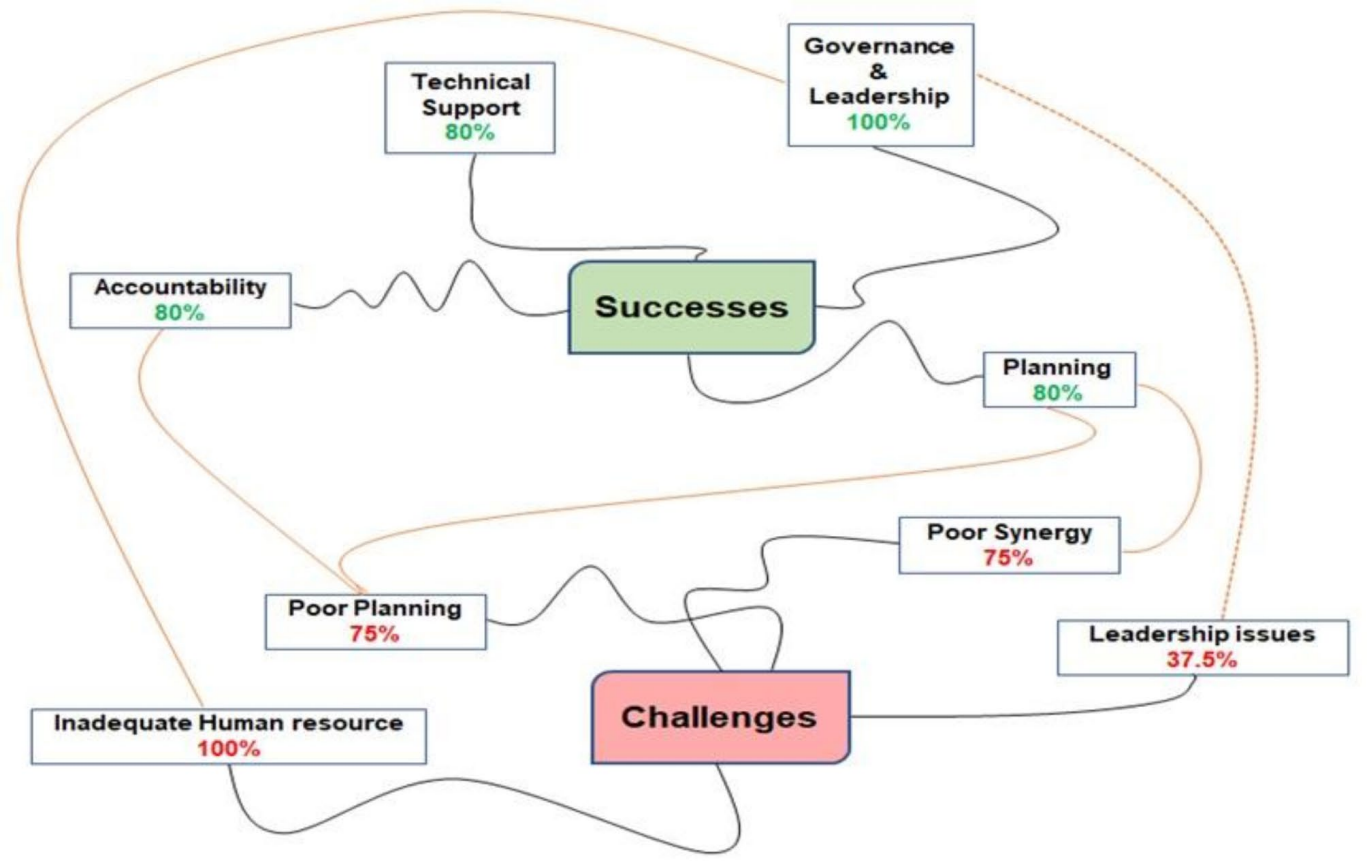
Thematic analysis and word mapping of the successes and challenges in the BHCPF implementation

From the thematic analysis and word mapping shown in Fig. 7, we identified four major factors that contributed to the successes of the BHCPF: governance and leadership (100%), technical support (80%), accountability (80%), and planning (80%). The major challenges identified during the BHCPF implementation across the six states since inception to date included: inadequate human resource (100%), poor planning (75%), poor synergy (75%), and leadership issues (37.5%).

When the BHCPF focal persons were ask:

What are the factors that have contributed to the successes recorded during the implementation of the BHCPF at the State, LGA and HF level?

…….*Exemplary leadership provided by the SPHCB, availability of funds to implement the program, strong BHCPF coordination structure in the State, which involves SOC, gateway forums and availability of WDCs at the community level, strong partner support from organizations such as Lafiya UK project, SCIDaR, CHAI, SOML and so on, and availability of BHCPF guidelines and accountability framework are some of the factors that contributed to the successes recorded during the implementation of the BHCPF in the state* - BHCPF focal person from one of the high performing state.

……. *Established forum where the LGA BHCPF FPs and the state share updates which lead to the quick completion of the Q1 and Q2 business plan development, instituted constant reminder from the state to the LGAs and HFs using established forum to disseminate information in real time, leveraged on the RI channel of communication to LGAs and HFs to also communicate to health facilities for prompt actions, leveraged the integrated supportive supervision to continue to provide supervision and monitoring of its implementation* - BHCPF focal person from one of the high performing state.

What are the challenges that affected successful BHCPF implementation at the State, LGA and HF level?

……. *Some of the challenges that affected the successful BHCPF implementation at the State, LGA and HF level included poor coordination and oversight from the BHCPF FPs due to conflicting priorities, delay in submission of business plan due to security situation and bad terrain, constant change of data tools used by BHCPF HFs from the national such as the change in the business plan and retirement tools, and poor planning by health facilities as evident in their business plans* - BHCPF focal person from one of the poor performing state.

……. *We encountered several challenges which bordered around centralized state-level business plan development, underdeveloped capacity of the HF staff, protracted health facility retirement finalization process (retirement submission is a prerequisite for fund disbursement), centralized state-level retirement process (top - bottom); absence of tools at HF level (retirement reports are often shared orally or in hand-written formats), irregular supervisory schedule; Unavailability of appropriate checklist, suboptimal conduct of BHCPF gateway and PIU meetings; poor synergy between BHCPF gateways, non-involvement of partners and the finance working group in BHCPF activities contributes to slow execution of activities and poor technical capacities* - BHCPF focal person from one of the poor performing state.

## Discussion

### NPHCDA-BHCPF performance

The development of a comprehensive rollout plan was a crucial step in the initial planning phase of the Basic Health Care Provision Fund (BHCPF) program. The establishment of functional primary healthcare boards across the six states involved in the program created a structure for effective coordination at the outset. This ensured a smooth implementation of activities in each state. Effective planning ensures the strategic allocation and utilization of resources, optimizing the program initiatives [34–36], while the coordination team having the right technical support provides the necessary expertise to design and implement robust systems tailored to the specific needs of the program [37, 38]. 

To equip the healthcare workers with the necessary knowledge and skills to effectively administer the BHCPF program, there were state-level training and capacity-building programs. These programs employed a standard Training-of-Trainers (TOT) approach for State Primary Health Care Development Agency/State Primary Health Care Board (SPHCDA/SPHCB) staff. Additionally, cascade training was provided for Primary Health Center (PHC) health workers, Ward Development Committee (WDC) members, and Local Government Health Authority (LGHA) supervisory staff. The training played a crucial role in enhancing the capacity of stakeholders at various levels, including the state, local government areas (LGAs), wards, and healthcare facilities. This empowerment enabled PHC staff to apply the acquired knowledge in developing work plans. These plans, in turn, served as the foundation for fund disbursement and retirement processes at the conclusion of each quarter. Capacity building strengthens the skills and capabilities of the healthcare workers fostering a sustainable and efficient program delivery at all levels [39, 40]. 

The implementation of the Basic Health Care Provision Fund (BHCPF) in Northern Nigeria, specifically through the NPHCDA gateway, varied considerably across the six states examined in the source. While some states demonstrated commendable efficiency and effectiveness, others grappled with significant challenges, ultimately impacting the program’s overall impact.

### Planning and preparation

The high performing state particularly Bauchi and Sokoto, exhibited a proactive approach to planning and preparation. For instance, Bauchi’s early completion of accreditation for all ward PHCs, attributed to robust technical and financial support, set the stage for smoother fund disbursement. Sokoto, by leveraging early preparatory activities, also achieved 100% accreditation by 2022. High-performing states generally excelled in developing and submitting business plans in a timely manner, contributing to efficient fund allocation and utilization. In contrast, states like Borno, Yobe, and to an extent, Sokoto, faced delays in the accreditation process, primarily due to contextual challenges. Borno’s progress was hampered by security issues and difficult terrain, leading to late business plan submissions. Yobe struggled with inadequate staff capacity, while Sokoto’s capacity constraints in business plan development resulted in approval delays. A common thread across most states was the difficulty in the timely review and finalization of business plans, often attributed to inadequate planning, multiple review iterations, limited technical expertise at the PHC level, and delays at the state level. The need for planning cannot be overemphasised as it arises from the necessity to balance the available resources and the resources needed to address the perceived health needs that face health systems [41].

### Fund disbursement

The efficiency in fund disbursement was a defining characteristic of high-performing states. Kano and Borno consistently disbursed funds without disruptions from Q3 2021, mainly due to streamlined processing of funding requests and prompt document submission. Bauchi’s success was linked to pre-existing robust financial systems. On the other hand, states facing implementation challenges experienced delays in disbursing funds. Borno and Kano, for instance, did not disburse funds in the initial quarters of implementation due to delays in start-up activities. Sokoto encountered significant delays attributed to bottlenecks in business plan development and late retirement of funds from previous quarters. Studies suggests that improved technical efficiency (that is, how to maximise output for a given level of inputs) and allocative efficiency (that is how to increase outputs through a better distribution and composition of inputs) is a perquisite for addressing programming inefficiencies; and most addressed through better policy and fiscal responsibility [42–44].

### Fund retirement

Bauchi, Kaduna, and Yobe excelled in the timely retirement of funds, indicating efficient utilization and proper financial management. These states likely benefited from established protocols, trained personnel, and effective monitoring mechanisms. Borno, Kano, and Sokoto struggled to complete the fund retirement process within the stipulated timeframe, suggesting potential weaknesses in financial reporting, accountability practices, or both. The source points to a lack of technical know-how among LGHA and healthcare facility teams as a contributing factor to these challenges.

### Governance and coordination

High-performing states like Kaduna and Kano benefited from well-structured coordination mechanisms. Kaduna’s effective leadership, coupled with the availability of clear BHCPF guidelines, facilitated a smoother implementation process. Proper coordination ensures that organizational processes and activities are well facilitated, which helps to reduce overlaps and inconsistencies [45, 46]. Kano’s success was attributed to strong commitment from key state actors, efficient information dissemination channels, and the reactivation of essential committees. However, it’s important to note that even high-performing states encountered some difficulties in consistently holding State Oversight Committee meetings. Challenges in coordination and governance were particularly noticeable in states like Borno and Yobe, where State Oversight Committee meetings were frequently not held. Additionally, inconsistent supportive supervision, particularly in states like Sokoto, where partner and state finance working groups were disengaged, further exacerbated the challenges in these states. Supportive supervision is a key component of health system strengthening efforts aimed at improving outcomes [47]. Research indicates that supportive supervision plays a crucial leadership role in ensuring the continuous improvement of health programs and maintaining performance during crises [48, 49].

### Factors contributing to current successes and barriers

Effective planning was identified as a crucial element for the success of BHCPF activities. For instance, Bauchi’s ability to leverage technical and financial support facilitated the establishment of operational PHCs in every ward. Similarly, in Borno, the use of existing social media and physical meeting platforms expedited plan development. Kaduna’s strong leadership and adherence to BHCPF guidelines streamlined the planning process. In Sokoto, early preparatory activities resulted in a 100% accreditation rate for PHC facilities by 2022. These are instances that highlights how thorough planning, supported by resources and effective communication, directly contributes to achieving desired program outcomes. However, there were notable challenges during the planning phase, which hindered the timely and complete implementation of BHCPF activities across different states. Notably, security concerns and challenging terrain in Borno led to delayed business plan submissions. Yobe’s progress was hampered by limited staff capacity, while Sokoto experienced approval delays due to inadequate capacity in developing business plans. These challenges underscore the importance of addressing contextual factors and building capacity at the state level to ensure the timely execution of BHCPF activities.

There was a strong link between functionality of existing systems and the success of fund disbursement during transition to financial management. States which already had existing financial management systems, like Bauchi’s with already mature financial systems for RI, demonstrated greater financial management outcomes in the disbursement of funds. The presence of experienced state staff, coupled with a robust accountability framework, were drivers for efficient fund disbursement. Conversely, the lack of technical expertise and capacity gaps in financial management were significant impediments to successful implementation noted in poor performing states. The poor performing states experienced challenges such as, delayed business planning and fund disbursement, as well as difficulties in the retirement process. This highlights the need for financial management capacity building activities and the establishment of robust accountability mechanisms to ensure the funds are effectively and transparently utilized.

The study emphasizes the role of governance and coordination in the successful implementation of the BHCPF, as the level of commitment of the coordinating structures are known to influence program implementation. States like Kano, which benefited from committed key officials, effective communication channels, and strong coordination structures, experienced enhanced governance and coordination. However, challenges such as conflicting priorities among stakeholder and inadequate secretariat functionality negatively impacted the frequency and effectiveness of coordination meetings in poorer performing states. This underscores the importance of strong leadership, dedicated coordination structures, and effective communication channels for successful program implementation.

Supportive supervision as a component of monitoring and evaluation significantly contributed to the success of the program. States such as Bauchi and Yobe, which achieved 100% in their planned supervisory visits to BHCPF facilities, suggested that to a large extent, their success was attributed to strong partner support, capacity building for supervisory staff, and effective supervision. Conversely, the absence of a structured supervisory schedule, inadequate capacity building, and insufficient funding for supervision activities negatively impacted the performance in states with poor ratings. This demonstrates the need for well-structured supervision plans, adequate funding, and capacity building for supervisory staff to ensure effective program implementation.

## Conclusion

The success of implementing the BHCPF across Nigerian states hinges on key factors. Early and strategic planning, technical proficiency in human resources, functionality of existing systems, cooperation between the government and partner organizations, and effective management and supervision are crucial determinants.

Some of the identified poor performing states had similar drawbacks hinged on conflicting priorities within the leadership structure, human resources’ technical capacity, which required competent health program managers and trained healthcare professionals. There is also the need to create an enabling environment that support their functions. Efficient program and financial management systems are vital for effective resource allocation, utilisation, accountability.

It is also important to call out how significant the role of collaboration between the government and partners was in the program implementation and accountability. This synergy enables resource pooling, information sharing, and coordinated efforts for effective healthcare programs. Effective management and supervision, with clear goals and progress monitoring, ensure optimal functioning of the primary healthcare system, allowing for necessary adjustments.

### Recommendations/implications for program improvement

Based on the observed successes and challenges of the BHCPF implementation in the six MoU states, the following recommendations are proposed to optimize the program performance in future:

- Channel resources to optimize the outstanding healthcare facilities (at least 1 per ward) toward accreditation for the BHCPF implementation to ensure scale across the states.
- Integrate financial management systems such that the BHCPF leverages functional systems including those established by the NSHIP and platforms such as the PHC MoUs to ensure system efficiency and streamline the account management processes at lower levels.
- Train/retrain state PIU members and LGHA officers on the standard protocols for BHCPF because these key stakeholders are integral to attaining the desired goals of the BHCPF and broader health sector reform efforts.
- Capacitate health workers at the PHCs by intensifying supportive supervision and conducting on-the-job training for the health facility staff on the development of business plans, funds management, retirement processes, and other basic skills.
  - ◦ The states might consider a refresher training at specified intervals, for example, biannually.
  - ◦ Implementers might also consider refocusing supervisory frameworks to prioritize on-the-job mentorship for health workers along with traditional performance monitoring.
  - ◦ Leverage existing supportive supervision structures and partner resources (for example, Integrated Supportive Supervision) to conduct these supervisory checks at the lower level.
- Design an effective M&E tool for efficient data collection and collation across all levels and provide Data Delivery support to coordination structures such as the SOC and Gateway forum.
- Support the BHCPF retirement process through the definition of stringent accountability measures and establishment of reminder systems for submission of LGA and health facility retirement documents.
- Improve collaboration and reduce bureaucratic bottlenecks that exist between the SPHCDAs, SHCMAs, and DMAs.
- Optimize staff capacity needs by exploring interdepartmental staff exchanges to leverage staff outside the PIU during peak periods of work (such as business plan reviews and retirement collation). Stakeholders should also advocate to the leadership of the SPHCB/DAs to optimize staff strength, capacity, and retention.

In a broader setting, the knowledge gained from the operational and programmatic experiences of states implementing the BHCPF is valuable for other LMICs. This should serve as a guide to navigate potential hurdles associated with PHC financing, by capitalizing on successful strategies and tailoring their approaches to suit their healthcare system dynamics and the specific needs of their populations. The information in this research is a valuable resource for LMICs aiming to enhance their PHC systems through the adoption of similar frameworks, ultimately contributing to more effective and context-appropriate healthcare financing strategies.

### Implication for policy

From a policy perspective, the study suggests that policymakers prioritize human resource for health (HRH) development by investing in training, improving salary structure and benefits, and creating a supportive work environment to attract and retain skilled healthcare professionals. They should also promote decentralized financial management systems by empowering local government health authorities and healthcare facilities with greater financial autonomy, which will potentially expedite fund disbursement and utilization. Policy makers are encouraged to develop context-specific strategies, which recognizes the varied challenges across the states. These policies should be capable of addressing specific local contexts, including security concerns and geographical barriers.

The policy makers and implementers should put systems in place to foster strong partnerships – as collaboration between government, partner organizations, and local communities is essential for effective implementation and sustainable healthcare improvements. By addressing these interconnected factors, policymakers and program implementers can work towards a more equitable and effective healthcare system that reaches those most in need.

### Limitations of the study

One important limitation of this study is its exclusive focus on six specific Nigerian states: Bauchi, Borno, Yobe, Kaduna, Kano, and Sokoto, which are all in the northern part of the country. While these states offer valuable insights into the implementation of the Basic Health Care Provision Fund (BHCPF), the findings may not be fully generalizable to other regions or countries with different healthcare systems, socio-economic conditions, or cultural contexts. The study’s applicability beyond the selected states may be constrained by variations in healthcare systems and policies. Additionally, the dynamic nature of healthcare practices suggests that changes post-study could influence the continued relevance of the findings over time.

## Supplementary Information

Supplementary Material 1.

## Data Availability

The datasets used and/or analyzed during the current study are available from the corresponding author on reasonable request.

## Abbreviations

BHCPF: Basic Health Care Provision Fund
BMPHS: Basic Minimum Package of Health Services
CHIPS: Community Health Influencers, Promoters, and Services
CRF: Consolidated Revenue Fund
DMA: Drug Management Agency
HF: Health Facility
KII: Key Informant Interview
LGA: Local Government Area
LGHA: Local Government Health Authority
LMIC: Low- and Middle-Income Country
M&E: Monitoring and Evaluation
MoU: Memorandum of Understanding
NCDC: Nigeria Centre for Disease Control
NEMTC: National Emergency Medical Treatment Committee
NHAct: National Health Act
NHIS: National Health Insurance Scheme
NSHIP: Nigeria States Health Investment Project
SHCMA: State Contributory Healthcare Management Agency
SOC: State Oversight Committee
SPHCMB/DA: State Primary Health Care Management Board / State Primary Health Care Development Agency
TOT: Training-of-Trainers
UHC: Universal Health Coverage
WDC: Ward Development Committee
WHO: World Health Organization
