# Supplementary material for: Evaluating the implementation of the National Primary Health Care Development Agency (NPHCDA) gateway for the Basic Healthcare Provision Fund (BHCPF) across six Northern states in Nigeria
Source: BMC Health Serv Res. 2024 Nov 14;24:1404. doi: 10.1186/s12913-024-11867-3 (PMC11566299; doi:10.1186/s12913-024-11867-3)
Supplement: Supplementary file 1 — Supplementary Material 1. [file 12913_2024_11867_MOESM1_ESM.docx]

**Table S1:** Distribution of wards in the States and HF accredited for BHCPF

| **State** | **Total # of wards** | **# of BHCPF HFs (%)** |
| --- | --- | --- |
| **Bauchi** | 323 | 323 (100) |
| **Borno** | 311 | 121 (38.9) |
| **Kaduna** | 255 | 254 (99.6) |
| **Kano** | 484 | 351 (72.5) |
| **Sokoto** | 244 | 244 (100) |
| **Yobe** | 178 | 158 (88.8) |
| **Total** | 1795 | 1451 (80.8) |

**Table S2:** Checklist for status of roll-out activities of the NPHCDA gateway

| **S/N** | **Roll-out activities** | **Status** |
| --- | --- | --- |
| 1 | Establishment and effective functioning of a State Primary Health Care Board or Agency and LGHAs in line with the PHCUOR policy |  |
| 2 | Open a TSA account with the Central Bank titled ‘SPHCDA/MB BHCPF’ |  |
| 3 | Show evidence of contributions of the state and LGHA 25% counterpart funding for the previous year |  |
| 4 | Identify at least 1 (one) functional health facility in each ward |  |
| 5 | Secure approval from HCH for selected health facilities |  |
| 6 | Conduct a baseline assessment to determine the status of the PHC system and priority health needs across the state |  |
| 7 | Determine a final listing of at least 1 (one) PHC per ward based on findings from assessments conducted |  |
| 8 | Conduct state level training and capacity building using basic training-of-trainers (TOT) method for SPHCDA/SPHCB |  |
| 9 | Conduct cascade training and capacity building using basic TOT method for PHC HWs, WDC members and LGHA supervisory staff |  |
| 10 | Develop PHC annual quality improvement plans that shall inform the quarterly business plans |  |
| 11 | Disburse funds to the PHC facility account following review and approval of annual QIPs |  |

*Source: BHCPF 2020 National guideline*

**Table S3:** Checklist for quantitative assessment of BHCPF implementation status

| **Focus** | **Indicators** | **Data sources** |
| --- | --- | --- |
| Planning | - Number of ward PHC facilities enlisted for BHCPF in the State | - BHCPF facility list |
|  | - Proportion of BHCPF facilities that submitted business plans for the quarter | - PHC business plans |
|  | - Proportion of BHCPF facilities that submitted business plans for the quarter on time |  |
|  | - Proportion of BHCPF facilities with approved business plans |  |
| Disbursement | - Proportion of quarterly BHCPF funds disbursed from National to the State | - Financial reports |
|  | - Proportion of BHCPF facilities that received funds for the quarter |  |
| Retirement | - Proportion of BHCPF facilities that submitted complete retirement documents |  |
|  | - Proportion of BHCPF facilities that submitted complete retirement documents on time |  |
| Governance and coordination | - Proportion of meetings held by the BHCPF State Oversight Committee (SOC) | - Meeting notes - Attendance sheet - Action point trackers |
|  | - Proportion of meetings held by the Gateways Forum |  |
|  | - Proportion of meetings held by the BHCPF PIU |  |
| Supervision | - Number of BHCPF facilities planned to be visited by the state supportive supervisory team | - Supervision plans and reports |
|  | - Proportion of eligible PHC centers supervised on BHCPF |  |

*Source: BHCPF 2020 National guideline*

**Table S4:** Key Informant Interview (KII) questionnaire guide

| **Instruction: Conduct a physical KIIs with key BHCPF PUI leads in the State** |
| --- |
| What are the factors that have contributed to the successes recorded during the implementation of the BHCPF at the State, LGA and HF level? (Please provide at least 5 success factors) |
| 1 |
| 2 |
| 3 |
| 4 |
| 5 |
|  |
| What are the challenges that affected successful BHCPF implementation at the State, LGA and HF level? (Please provide at least 5 challenges) |
| 1 |
| 2 |
| 3 |
| 4 |
| 5 |
|  |
| What are the recommendations you propose to address the challenges identified? |
| (Please provide at least 5 recommendations) |
| 1 |
| 2 |
| 3 |
| 4 |
| 5 |
|  |
